# Supplementary material for: Antibody Response to the BA.5 Bivalent Vaccine Shot: a Two-Year Follow-Up Study following Initial COVID-19 mRNA Vaccination
Source: Microbiol Spectr. 2023 May 16;11(3):e01316-23. doi: 10.1128/spectrum.01316-23 (PMC10269437; doi:10.1128/spectrum.01316-23)
Supplement: Supplemental file 1 — Supplemental material. Download spectrum.01316-23-s0001.pdf, PDF file, 0.33 MB [file spectrum.01316-23-s0001.pdf]

Antibody Response to the BA.5 Bivalent Vaccine Shot: a Two-Year Follow-up Study following Initial COVID-19 mRNA Vaccination

Yosuke Hirotsu[1]*, Hiroki Sugiura[2], Mika Takatori[3], Hitoshi Mochizuki[1,4,5] and Masao Omata[5,6]

[1]Genome Analysis Center, Yamanashi Central Hospital, Kofu, Japan
[2]Division of Clinical Biochemistry and Immunology, Yamanashi Central Hospital, Kofu, Japan
[3]Division of Infection Control and Prevention, Yamanashi Central Hospital, Kofu, Japan
[4]Central Clinical Laboratory, Yamanashi Central Hospital, Kofu, Japan
[5]Department of Gastroenterology, Yamanashi Central Hospital, Kofu, Japan
[6]The University of Tokyo, Tokyo, Japan

***Corresponding author:** Yosuke Hirotsu, Genome Analysis Center, Yamanashi Central Hospital, Kofu, Japan
Email: hirotsu-bdyu@ych.pref.yamanashi.jp
Tel: +81-55-253-7111, Fax: +81-55-253-8011

SUPPLEMENTARY INFORMATION

MATERIALS AND METHODS

Participants and Samples

Written informed consent was obtained from all HCWs (n=46) who enrolled in this study. The HCWs received monovalent mRNA vaccine (Pfizer-BioNTech, n=46) for their first to third doses (V1-V3), monovalent mRNA vaccine (Moderna, n=31; Pfizer-BioNTech, n=15) for their fourth dose (V4), and BA.5-adapted bivalent mRNA vaccine (Pfizer-BioNTech) for their fifth dose ($V5_{bivalent}$) (Table 1). The cohort included three HCWs who had been previously infected with SARS-CoV-2, while 43 were infection-naïve and had no history of infection.

Peripheral blood samples were serially drawn from the HCWs 11 times, including just before vaccination (baseline), 3 weeks after V1 (3W), 1 week after V2 (4W), 2 weeks after V2 (5W), 3 weeks after V2 (6W), 4 weeks after V2 (7W), 9 weeks after V2 (12W), 27 weeks after V2 (30W), 11 weeks after V3 (52W), just before $V5_{bivalent}$ (100W), and 2 weeks after $V5_{bivalent}$ (102W) (Supplementary Fig 1 and Supplementary Table 1).

Serological Analysis

We measured the levels of anti-S protein RBD antibody using the Elecsys Anti-SARS-CoV-2 S antibody test (Roche Diagnostics, Basel, Switzerland) on a cobas® 8000 automated platform (1). This assay utilizes the electrochemiluminescence immunoassay principle. For anti-S antibody, samples containing <0.8 unit/mL (U/mL) were considered negative, while those containing ≥0.8 U/mL were considered positive following the manufacturer's instructions.

SARS-CoV-2 Diagnostic Testing

Multiple molecular diagnostic platforms of nucleic acid amplification and antigen testing were used to diagnose SARS-CoV-2 infection. The diagnostic tests used were reverse transcription PCR (in accordance with the protocol developed by the National Institute of Infectious Diseases in Japan (2)), the FilmArray Respiratory Panel 2.1 test with the FilmArray Torch system (bioMérieux, Marcy-l'Etoile, France) (3), the Xpert Xpress SARS-CoV-2 test using Cepheid GeneXpert (Cepheid, Sunnyvale, CA) (4), and the Lumipulse antigen test with the LUMIPULSE G600II system (Fujirebio, Inc., Tokyo, Japan) (5, 6). All tests were performed with material obtained from nasopharyngeal swabs immersed in viral transport medium (Copan, Murrieta, CA).

Ethics Statement

The Institutional Review Board of the Clinical Research and Genome Research Committee of Yamanashi Central Hospital approved this prospective study (Approval No. C2019-30). Participation in the study was optional following informed consent. All study procedures were performed in accordance with the relevant guidelines and regulations and as set out in the Helsinki Declaration.

Statistical Analysis

The interquartile range (IQR) was calculated and statistical analyses were performed using R version 3.6.2 (http://www.r-project.org/). The following R packages were used for data cleaning, analysis, and visualization: ggplot2 (v3.3.5), dplyr (v1.0.7), tidyr (v1.1.3), patchwork (v1.1.1), lubridate (v1.9.0), rstatix (v0.7.1), gtsummary (v1.5.2), and flextable (v.0.7.0). The Wilcoxon rank sum test and paired Wilcoxon signed-rank test were used. To analyze the data by age group, the Wilcoxon rank sum test was used with the 20s group as the reference. The resulting p-values were adjusted for multiple comparisons using Bonferroni correction and reported as adjusted p-values (padj). Statistical significance was defined as P value < 0.05.

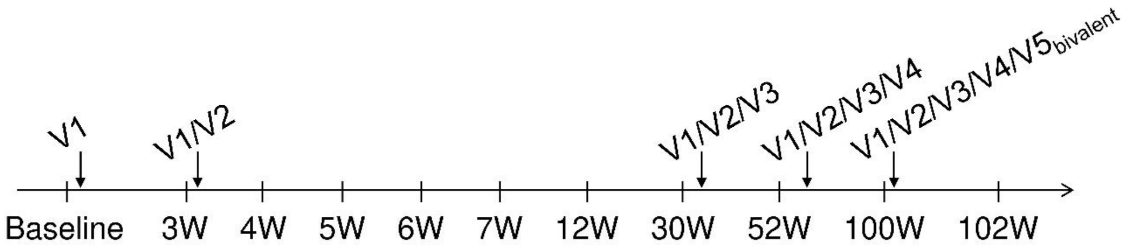

Supplementary Figure 1. Vaccination timing during the observation period

A total of 11 blood samples were collected from 46 healthcare workers at the following timepoints: just before vaccination (baseline), 3 weeks after the first dose (3W), 1 week after the second dose (4W), 2 weeks after the second dose (5W), 3 weeks after the second dose (6W), 4 weeks after the second dose (7W), 9 weeks after the second dose (12W), 27 weeks after the second dose (30W), 11 weeks after the third dose (52W), just before the fifth dose (100W), and 2 weeks after the fifth dose (102W). V1: first dose, V2: second dose, V3: third dose, V4: fourth dose, and $V5_{bivalent}$: fifth dose of the vaccine, which was a BA.5-adapted bivalent vaccine.

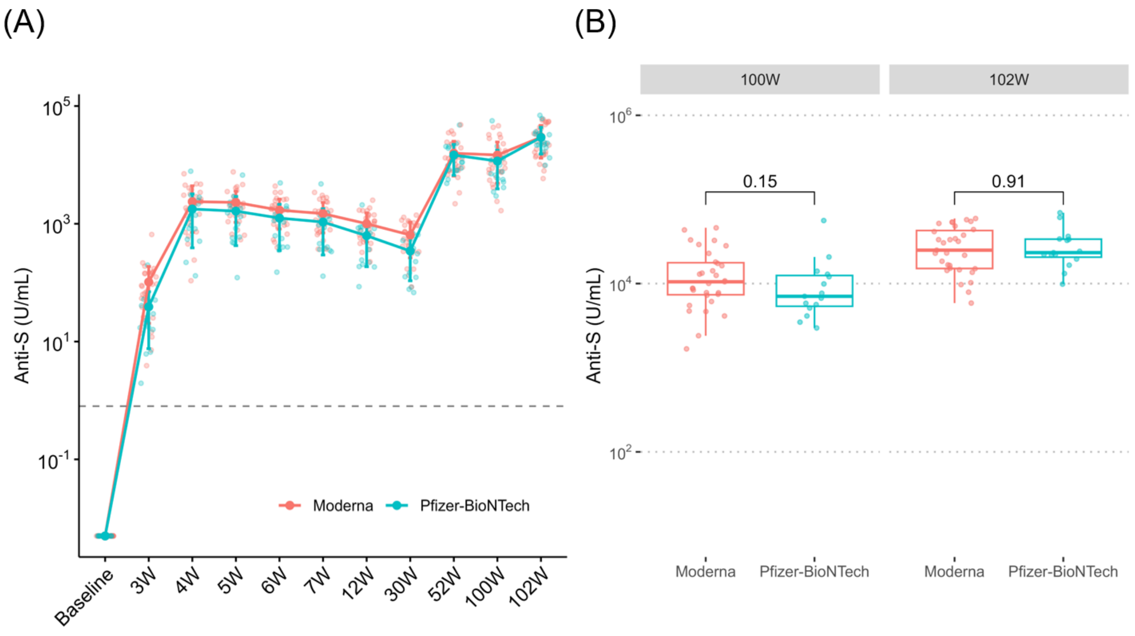

Supplementary Figure 2. Spike antibody levels and vaccine type of the fourth vaccination

The fourth vaccination was administered using vaccines supplied by Moderna (n=31) or Pfizer-BioNTech (n=15). (**A**) The line plot shows the mean and standard deviation of spike antibody levels for each vaccine type. Each dot represents the antibody level of an individual HCW. The dashed line represents the cutoff value of spike antibody titer at 0.8 U/mL. (**B**) The box plot shows the spike antibody levels at 100W and 102W for each vaccine type. Statistical analysis was performed using the Wilcoxon rank sum test.

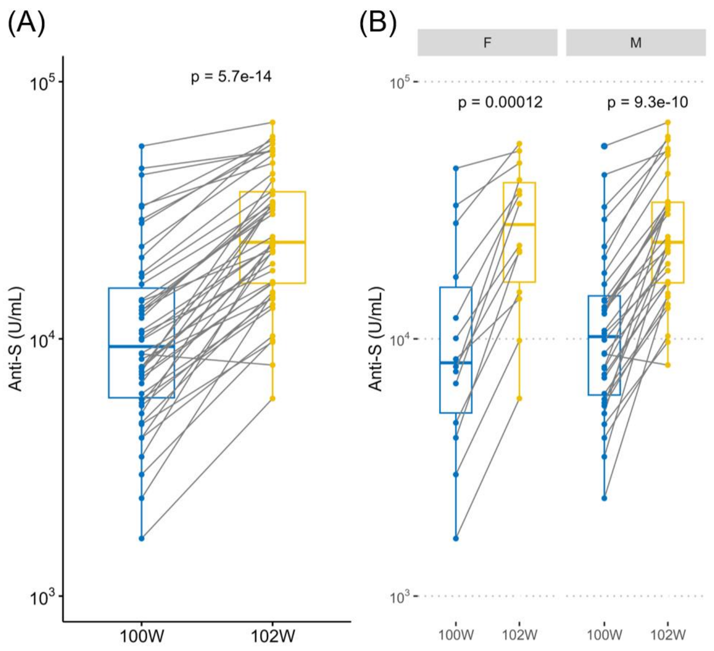

Supplementary Figure 3. Spike antibody levels before and after administration of the BA.5-adopted bivalent vaccine

Spike antibody levels (U/mL) at 100W (before $V5_{bivalent}$) and 102W (2 weeks after V5 $_{bivalent}$) for (**A**) all HCWs and (**B**) by sex. Box plots show the data before (100W) and two weeks after (102W) administration of the BA.5 adapted bivalent vaccine. Each box indicates the interquartile range (top: the third quartile; bottom: the first quartile) with a horizontal line indicating the median. Statistical analysis was performed by paired Wilcoxon signed-rank test.

Supplementary Table 1. Vaccination information

Timepoint	Vaccination count	Vaccination Timing
Baseline	-	Unvaccinated
3W	V1	3 weeks after V1
4W	V1/V2	1 weeks after V2
5W	V1/V2	2 weeks after V2
6W	V1/V2	3 weeks after V2
7W	V1/V2	4 weeks after V2
12W	V1/V2	9 weeks after V2
30W	V1/V2	27 weeks after V2
52W	V1/V2/V3	11 weeks after V3
100W	V1/V2/V3/V4	24-34 weeks after V4<br>Before $V5_{Bivalent}$
102W	V1/V2/V3/V4/$V5_{Bivalent}$	2 weeks after $V5_{Bivalent}$

W, weeks; V, vaccination.

Note: the timing of the administered vaccines: V1 at 0W, V2 at 3W, V3 at 41W, V4 between 66-76 weeks and V5bivalent at 100W.

References

1. **Hirotsu Y, Amemiya K, Sugiura H, Shinohara M, Takatori M, Mochizuki H, Omata M.** 2021. Robust Antibody Responses to the BNT162b2 mRNA Vaccine Occur Within a Week After the First Dose in Previously Infected Individuals and After the Second Dose in Uninfected Individuals. Frontiers in Immunology **12**.
2. **Shirato K, Nao N, Katano H, Takayama I, Saito S, Kato F, Katoh H, Sakata M, Nakatsu Y, Mori Y, Kageyama T, Matsuyama S, Takeda M.** 2020. Development of Genetic Diagnostic Methods for Novel Coronavirus 2019 (nCoV-2019) in Japan. Jpn J Infect Dis **73:**304-307
3. **Hirotsu Y, Maejima M, Shibusawa M, Amemiya K, Nagakubo Y, Hosaka K, Sueki H, Mochizuki H, Tsutsui T, Kakizaki Y, Miyashita Y, Omata M.** 2020. Analysis of Covid-19 and non-Covid-19 viruses, including influenza viruses, to determine the influence of intensive preventive measures in Japan. J Clin Virol **129:**104543.
4. **Hirotsu Y, Maejima M, Shibusawa M, Natori Y, Nagakubo Y, Hosaka K, Sueki H, Amemiya K, Hayakawa M, Mochizuki H, Tsutsui T, Kakizaki Y, Miyashita Y, Omata M.** 2022. Direct comparison of Xpert Xpress, FilmArray Respiratory Panel, Lumipulse antigen test, and RT-qPCR in 165 nasopharyngeal swabs. BMC Infectious Diseases **22:**221.
5. **Hirotsu Y, Maejima M, Shibusawa M, Amemiya K, Nagakubo Y, Hosaka K, Sueki H, Hayakawa M, Mochizuki H, Tsutsui T, Kakizaki Y, Miyashita Y, Omata M.** 2021. Prospective Study of 1,308 Nasopharyngeal Swabs from 1,033 Patients using the LUMIPULSE SARS-CoV-2 Antigen Test: Comparison with RT-qPCR. International Journal of Infectious Diseases **105:**7-14.
6. **Hirotsu Y, Maejima M, Shibusawa M, Nagakubo Y, Hosaka K, Amemiya K, Sueki H, Hayakawa M, Mochizuki H, Tsutsui T, Kakizaki Y, Miyashita Y, Yagi S, Kojima S, Omata M.** 2020. Comparison of Automated SARS-CoV-2 Antigen Test for COVID-19 Infection with Quantitative RT-PCR using 313 Nasopharyngeal Swabs Including from 7 Serially Followed Patients. International Journal of Infectious Diseases **99:**397-402.
